# Robot-assisted pelvic floor reconstructive surgery: an international Delphi study of expert users

**DOI:** 10.1007/s00464-023-10001-4

**Published:** 2023-03-23

**Authors:** Tommaso Simoncini, Andrea Panattoni, Mustafa Aktas, Jozef Ampe, Cornelia Betschart, Alexander L. A. Bloemendaal, Stephan Buse, Giuseppe Campagna, Marta Caretto, Mauro Cervigni, Esther C. J. Consten, Hugo H. Davila, Jean Dubuisson, Eloy Espin-Basany, Bernardina Fabiani, Jean-Luc Faucheron, Andrea Giannini, Brooke Gurland, Dieter Hahnloser, Ralf Joukhadar, Paolo Mannella, Liliana Mereu, Jacopo Martellucci, Guillaume Meurette, Maria Magdalena Montt Guevara, Carlo Ratto, Barry A. O’Reilly, Christl Reisenauer, Eleonora Russo, Steven Schraffordt Koops, Shahab Siddiqi, Alessandro Sturiale, Gabriele Naldini

**Affiliations:** 1 Division of Obstetrics and Gynecology, Department of Clinical and Experimental Medicine, University of Pisa, Pisa, Italy; 2 Division of Obstetrics and Gynecology, Maasstad Hospital, Rotterdam, The Netherlands; 3 Department of Urology, AZ Sint-Jan Bruges Hospitals, Brugge, Belgium; 4 Department of Gynecology, University Hospital of Zurich, University of Zurich, Zurich, Switzerland; 5 Department of Surgery, Reinier de Graaf Hospital, Delft, The Netherlands; 6 Department of Urology and Urologic Oncology, Alfried Krupp Hospital, Essen, Germany; 7 Division of Urogynecology and Pelvic Floor Reconstructive Surgery, Department of Women and Child Health, University Hospital A. Gemelli IRCCS, Catholic University of the Sacred Heart, Rome, Italy; 8 Department of Urology, La Sapienza University-Polo Pontino ICOT, Latina, Italy; 9 Department of Surgery, Meander Medical Center, Amersfoort and Department of Surgery, University Medical Center Groningen, Groningen, The Netherlands; 10 Cleveland Clinic Indian River Hospital, Florida State University, College of Medicine, Tallahassee, FL USA; 11 Department of Pediatrics, Gynecology, and Obstetrics, Geneva University Hospitals and University of Geneva, Geneva, Switzerland; 12 Unidad de Cirugía Colorrectal, Servicio de Cirugía General, Hospital Valle de Hebron, Universitat Autonoma de Barcelona, Barcelona, Spain; 13 Proctology and Pelvic Floor Clinical Center, Cisanello University Hospital, Pisa, Italy; 14 Colorectal Surgery Unit, Visceral Surgery and Acute Care Surgery Department, Grenoble Alps University Hospital, Grenoble, France; 15 Division of Colorectal Surgery, Department of Surgery, Stanford University School of Medicine, Stanford, CA USA; 16 Department of Visceral Surgery, University Hospital Lausanne, Lausanne, Switzerland; 17 Department of Obstetrics and Gynecology, University of Wuerzburg, Würzburg, Germany; 18 Department of Obstetrics and Gynecology, Cannizzaro Hospital, Catania, Italy; 19 Department of General, Emergency and Minimally Invasive Surgery, Careggi University Hospital, Florence, Italy; 20 Digestive and Endocrine Surgery Clinic, IMAD, CHU de Nantes, Hôtel Dieu, Nantes Cedex, France; 21 Proctology Unit, Fondazione Policlinico Universitario Agostino Gemelli IRCCS, Università Cattolica del Sacro Cuore, Rome, Italy; 22 Department of Obstetrics and Gynecology, Cork University Maternity Hospital, Cork, Ireland; 23 Department of Obstetrics and Gynecology, University Hospital Tuebingen, Tuebingen, Germany; 24 Department of Gynecology, Meander Medical Center, Amersfoort, The Netherlands; 25 Broomfield Hospital, Court Road, Chelmsford, UK

**Keywords:** Pelvic floor reconstructive surgery, Robotic surgery, Pelvic organ prolapse, Rectal prolapse, Delphi consensus

## Abstract

**Background:**

Robotic surgery has gained popularity for the reconstruction of pelvic floor defects. Nonetheless, there is no evidence that robot-assisted reconstructive surgery is either appropriate or superior to standard laparoscopy for the performance of pelvic floor reconstructive procedures or that it is sustainable. The aim of this project was to address the proper role of robotic pelvic floor reconstructive procedures using expert opinion.

**Methods:**

We set up an international, multidisciplinary group of 26 experts to participate in a Delphi process on robotics as applied to pelvic floor reconstructive surgery. The group comprised urogynecologists, urologists, and colorectal surgeons with long-term experience in the performance of pelvic floor reconstructive procedures and with the use of the robot, who were identified primarily based on peer-reviewed publications. Two rounds of the Delphi process were conducted. The first included 63 statements pertaining to surgeons’ characteristics, general questions, indications, surgical technique, and future-oriented questions. A second round including 20 statements was used to reassess those statements where borderline agreement was obtained during the first round. The final step consisted of a face-to-face meeting with all participants to present and discuss the results of the analysis.

**Results:**

The 26 experts agreed that robotics is a suitable indication for pelvic floor reconstructive surgery because of the significant technical advantages that it confers relative to standard laparoscopy. Experts considered these advantages particularly important for the execution of complex reconstructive procedures, although the benefits can be found also during less challenging cases. The experts considered the robot safe and effective for pelvic floor reconstruction and generally thought that the additional costs are offset by the increased surgical efficacy.

**Conclusion:**

Robotics is a suitable choice for pelvic reconstruction, but this Delphi initiative calls for more research to objectively assess the specific settings where robotic surgery would provide the most benefit.

Pelvic floor reconstructive surgery (PFRS) is an emerging field of application for robotic platforms, with expert users experiencing advantages from the improved vision and dexterity, which they assert are particularly useful during abdominal procedures for prolapse. Clarity of vision in narrow anatomical areas and facilitated dissection and stitching in the deep pelvis are often indicated as key reasons why surgeons choose a robotic approach to perform abdominal pelvic organ prolapse (POP) reconstructive procedures. Robotic assistance has been used for all the described abdominal techniques, including sacral suspension, lateral suspension, rectal suspension, and para-vaginal repair, as well as anti-incontinence procedures [1–6].

Evidence corroborating the perception that robotics makes PFRS easier or more effective is so far missing, including safety, reproducibility, and efficacy in the hands of surgeons with different levels of experience. Nonetheless, studies have suggested that robotic surgery reduces the conversion rate to laparotomy and shortens the learning curve for complex procedures, such as colposacropexy [7–9]. Expert users of the robot believe that this platform may facilitate the development and spread of PFRS procedures, with better functional outcomes and long-term success rates for the treatment of advanced or multicompartmental prolapses compared with traditional transvaginal or transperineal procedures [4, 10].

Improving the efficacy of PFRS would have a relevant impact because this surgery is performed in high numbers and repeat procedures due to relapses increase expenditures [11]. In this light, sustainability and cost-effectiveness of using the robot for POP surgery should be thoroughly assessed [12, 13]. These answers will apply to urologic, gynecologic, and proctologic procedures because of the similarities in terms of anatomical dissection, suspensive strategies, and technical challenges [6].

The current study uses a Delphi methodology to report the experience of expert users on the performance of the Da Vinci® robotic platform (Intuitive Surgical, Sunnyvale, CA) for PFRS. Our results represent the opinions of an international group of high-volume surgeons from various specialties on the role of robot-assisted PFRS.

## Materials and methods

The study is based on the Delphi process, a method of structured communication applied to reach a consensus on a specific topic with input from a panel of experts. The Delphi technique uses a multistage, self-completed questionnaire with individual feedback [14, 15].

### Selection of the participants

Between November 2020 and September 2021, 37 high-volume robotic surgeons with extensive experience in pelvic floor reconstruction from all over the World were invited to join this project. Urogynecologists, urologists, and colorectal surgeons with an interest in proctology were included. Candidates were identified primarily based on peer-reviewed publications on robotic PFRS procedures or because of involvement in robotic special interest groups from international scientific societies dedicated to pelvic floor disorders. Participant selection was performed by the organizers of the initiative (Tommaso Simoncini and Gabriele Naldini, with the aid of collaborators).

Upon invitation of the 37 candidates, 4 declined participation or did not answer and 7 accepted but did not complete either the first or the second round of questions and were therefore excluded. Nonresponders were evenly distributed in the different geographic areas and surgical specialties. Twenty-six experts completed all Delphi rounds and participated in the joint discussion during the final teleconference, where the results were presented. The number of participants is within the range that is considered appropriate for Delphi studies [15].

A scientific committee was formed at the beginning of the process and included the 2 organizers plus 5 surgeons who were representative of the various specialties, geographic distributions, and both sexes (Consten, Davila, Meurette, Reisenauer, and Schraffordt Koops). The professional characteristics of the experts involved in the Delphi process are described in Table 1.

**Table 1:** Descriptive analysis of the participating experts

Category	*N* = 26 (%)
Specialists	
Colorectal surgeons	11 (42)
Urologists	4 (16)
Gynecologists	11 (42)
Countries	
Italy	8 (29)
The netherlands	4 (15)
Germany	3 (12)
France	2 (8)
The USA	2 (8)
The united kingdom	1 (4)
Ireland	1 (4)
Spain	1 (4)
Belgium	1 (4)
Switzerland	3 (12)
No. of robotic pfrs per year (range)	
1–20	11 (42)
20–40	8 (31)
> 40	7 (27)
Experience of surgeon (years)	
1–2	1 (4)
3–4	5 (19)
> 5	20 (77)
Surgeons performing pfrs with other techniques	
Lps	15 (58)
No lps	11 (42)
Transvaginal	19 (73)
No transvaginal	7 (27)
Transanal	10 (38)
No transanal	16 (62)
Surgeons employed in a hospital in which there is a multidisciplinary team for pf disorders	
Yes	23 (88)
No	3 (12)

*LPS* laparoscopy, *PF* pelvic floor, *PFRS* pelvic floor reconstructive surgery

### The Delphi process

The Delphi process was initiated by identifying the research statements. This was done by the scientific committee through a series of teleconferences. A total of 64 questions and statements were formulated for the first round, divided into 5 categories: 8 questions related to surgeons’ characteristics and 56 general statements, indications, surgical technique, and future-oriented statements. The questionnaire was sent to the participants using Google modules. The participants were asked to vote using a binary Likert scale of 1 through 9 to express their degree of agreement with the statement (with 1 indicating strong disagreement and 9 strong agreement). The participants were also asked to provide a comment explaining their choice.

Upon completion of the first round, the scientific committee analyzed the responses. As predefined in the study setup, those statements for which 70% or more of the group declared disagreement (score of 1–3) or agreement (score of 7–9) were excluded from the second round. Similarly, statements for which the group showed no concordance (scatter distribution of scores) were considered areas of no consensus and were excluded. Those statements for which 60% to 70% of the group either agreed or disagreed were reanalyzed by the scientific committee by looking at the explanatory comments. The committee rephrased these statements for the second round.

The second questionnaire was composed of 20 statements, and the same procedure was followed for analysis. The Delphi statements and the level of agreement of round 1 and round 2 are listed in Table 2 and Table 3.

**Table 2:** First round of the Delphi process

Level of agreement (*N* = 26)	
General questions	
RS allows you to reduce operative times for the reconstruction of PF (meaning whole surgical time including docking and undocking as compared with open or standard LPS)	31%
RS allows better vision during PF reconstructive surgery (independent of the scope used)	96%
The range of motion and articulation of robotic instruments offer an advantage for PF reconstructive surgery	96%
The learning curve for reconstructive PF procedures is shorter with robot-assisted surgery than with traditional laparoscopic surgery	76%
It is not necessary to have expertise in traditional laparoscopy to perform robotic reconstructive PF surgery	27%
The use of the robot helps in transferring skills in PF reconstruction to other surgeons	85%
The use of the robot for PF reconstruction is safe	92%
RS facilitates a quick discharge of patients undergoing PF reconstruction	85%
Use of robot-assisted surgery could help increase the uptake of transabdominal PF reconstructive procedures	65%
RS could decrease the rates of intra- and postoperative complications associated with PF reconstruction	54%
The robot is less of a physical strain for my body in comparison to traditional LPS	85%
The use of RS makes the surgery easier so I can concentrate longer	65%
After a day of RS, I feel less tired than after a day of conventional surgery	73%
The robot allows me to perform more PF reconstructive procedures in the same day than with standard LPS	38%
A more widespread use of the robot would allow an increase in the number of PF reconstructive procedures and would decrease the waiting time to access surgery	46%
The organizational ‘fit’ into existing pathways and frameworks is a barrier to the widespread use of the robot	35%
Lack of acceptance and resistance to change from colleagues are still able to affect the widespread use of RS	50%
The collection of PROM/PREM data can facilitate the widespread use of robotics and highlight its multidimensional benefits (e.g., socioeconomic)	92%
Covid-19 is negatively affecting the widespread use of the robot for PF reconstruction	58%
I would recommend the use of robot-assisted surgery for PF reconstruction to other colleagues	88%
Training sessions and opportunities for inexperienced surgeon assistants are easily available in my center/hospital	65%
I feel close to the current real-world evidence literature, as it gives a realistic picture of benefits and limits of RS	50%
Indications	
I choose robotic surgery over standard LPS to perform PFRS in obese patients	85%
I choose robotic surgery over standard LPS to perform PFRS in patients with previous abdominal surgery	73%
I choose robotic surgery over standard LPS to perform PFRS in patients with advanced prolapse	77%
I choose robotic surgery over standard LPS to perform PFRS in patients with multicompartmental prolapse	81%
I choose robotic surgery over standard LPS to perform PFRS in patients with relapsed/recurrent prolapse	73%
I choose robotic surgery over standard LPS to perform PFRS in patients who underwent previous reconstructive surgeries with meshes	77%
I choose robotic surgery over standard LPS to manage patients with late complications associated with previous PFRS (erosions, abscesses, fistulas)	62%
I choose robotic surgery over standard LPS to perform PFRS in patients with previous ureteral injury	46%
I choose robotic surgery over standard LPS to perform PFRS due to near-infrared fluorescence (NIRF) imaging using indocyanine green dye (ICG)	19%
Surgical technique	
It is mandatory to use 4 robotic arms (1 optic arm + 3 assistant arms) to perform PF reconstructive procedures	27%
The use of advanced energy devices is needed for robot-assisted pelvic floor surgery	27%
The last generation of robotic tools (Da Vinci Xi, integrated table motion) has improved reconstructive PF surgery over the previous platforms	46%
Robotic single-port technology is potentially useful for PF robotic reconstruction	31%
The isolation of the sacral promontory is more precise and safer with the aid of the robot	70%
The robot allows the management of major vascular damage at the level of the sacral promontory	42%
The robot decreases the risk of nerve injuries at the level of the sacral promontory	54%
The robot decreases the risk of injuries of the pelvic ureters	38%
The robot simplifies the performance of hysterectomy in the context of PFRS	46%
The robot simplifies deep dissection between the rectum and the vagina	77%
Robot surgery simplifies deep dissection between the vagina and the bladder	69%
Robotic surgery simplifies dissection of the Retzius space	73%
The robot simplifies and increases the accuracy of suturing on the sacral promontory	92%
The robot simplifies and increases the accuracy of suturing on the posterior wall of the vagina	92%
The robot simplifies and increases the accuracy of suturing on the anterior wall of the vagina	73%
The robot simplifies and increases the accuracy of suturing in the Retzius space	73%
The robot simplifies and increases the accuracy of suturing on the anterior rectal wall	77%
The robot allows better positioning of meshes	62%
The modality of robot-assisted suturing and mesh positioning could decrease complications related to synthetic non-resorbable meshes	50%
Future-oriented questions	
Taking into consideration the costs and benefits of RS, PF reconstruction is a suitable indication for robotics for every patient	58%
Taking into consideration the costs and benefits of RS, PF reconstruction is a suitable indication for robotics only for selected patients	50%
If I were the manager of a hospital, I would approve a robotic program for PF reconstruction	77%
If I were a patient with a pelvic floor disorder, I would preferentially select a hospital with a robotic program	81%
The spread of the use of the robot for PF reconstruction may in future become a socioeconomic advantage (e.g., because of increased performance of transabdominal reconstructive procedures or possible decreases in relapses)	62%
Where do you see the future main developments of robotic technology for PFRS? (open question)	
▪Preoperative anatomical planning and surgical navigation applications to perform pelvic floor reconstruction (66%)	
▪The development of tactile feedback (66%)	
▪Availability of new energies applied to the robotic platform (17%)	
▪Development of new tools for pelvic floor reconstruction with the robot (52%)	
▪Development of new types of robots for pelvic floor reconstruction (34%)	
▪Applications of artificial intelligence or deep learning for pelvic floor reconstruction with the robotic platform (66%)	
▪Less or no need for an assistant surgeon (59%)	
▪Others: -Ultrasound-assisted robotic surgery -Ultra mini-invasive approach	

*LPS* laparoscopy, *PF* pelvic floor, *PFRS* pelvic floor reconstructive surgery, *PROM/PREM* patient-reported outcomes/patient-reported experience measure, *RS* robotic surgery

**Table 3:** Second round of the Delphi Process

Level of agreement (*N* = 26)	
General questions	
RS performed with the Da Vinci Xi platform may allow for reducing operative times versus standard LPS for the reconstruction of PF in complex surgical cases (please consider only the console time, without including docking and undocking)	88%
It is mandatory to have expertise in traditional LPS before starting to perform PFRS	19%
Gaining access to a robot in a practice where this was not available before would likely increase the number of transabdominal PFRS procedures (without considering the costs)	54%
RS could decrease the rates of complications associated with PF reconstruction in complex cases	62%
The presence of a dedicated center for RS with expert staff facilitates the use of the robot for non-oncologic applications such as PFRS	92%
There are still a lack of acceptance of RS for PF reconstruction within the surgical community	46%
Training sessions and opportunities for inexperienced surgeon-assistant trainees are important to perform PFRS	91%
The current literature does not yet correctly depict the advantages of robotics for PF reconstruction	62%
Surgical technique	
The use of the fourth robotic arm is not always mandatory to perform PF reconstructive procedures in simple cases	81%
The standard robotic monopolar and bipolar instruments of the intuitive Da Vinci Xi system are sufficient to perform PFRS	96%
The development of an effective single-port robotic platform is of interest for PFRS	73%
In case of major vascular damage at the level of the sacral promontory, surgical management with the robot is less effective than with standard LPS	8%
The identification of nerve bundles during PF reconstructive surgery is facilitated by the robot as compared with standard LPS	50%
RS decreases the time needed to perform supracervical hysterectomy during PFRS compared with standard LPS	31%
Suturing mesh on the anterior rectal wall is easier with the robot than with standard LPS	77%
Suturing mesh on the anterior rectal wall is safe with the robot	77%
Mesh placement is more precise with the use of robotic instruments as compared with standard LPS	73%
Future-oriented questions	
Taking into consideration the costs and benefits of PFRS, PF reconstruction with robotics is a suitable indication for patients requiring abdominal correction of POP	81%
Taking into consideration the costs of RS, PFRS should be limited to selected indications	50%
If a more widespread use of the robot in future will lead to more abdominal PF reconstructive procedures, this is likely to turn into socioeconomic advantages (e.g., because of decreased relapses and/or reoperations)	58%

*LPS *laparoscopy, *PF* pelvic floor, *PFRS* pelvic floor reconstructive surgery, *POP* pelvic organ prolapse, *RS* robotic surgery

The scientific committee then adjudicated the areas of consensus and of lack of agreement and performed subanalyses looking at how the opinions of the group varied based on the specialty of the surgeons, the surgeons’ experience with the use of the robot, surgeons’ annual case load, and on their habit of using also standard laparoscopy.

The third and concluding step of the Delphi process consisted of a face-to-face virtual meeting with all the participants, where the data were reviewed, and several comments were discussed and agreement with the analysis of the committee was verified.

### Data analysis

Categorical data are described as absolute frequency. Stratified analyses of responses to questions of the 2 Delphi rounds, as a dichotomized agreement, were performed with chi-square test (*χ*^2^), Fisher’s exact test, and *χ*^2^ test for trend. Significance was set at 0.05. All analyses were performed with SPSS technology v.27 (SPSS Inc., Chicago, IL).

## Results

The first questions in the first Delphi round provided a description of the participants (Table 1). Participants were representative of the 3 specialties and of different geographic areas and health systems. Most of the participants had been using the robot to perform PFRS for more than 3 years, with a mean of more than 20 procedures per year. Half of the group performed PFRS via both laparoscopic and robotic techniques and a majority also performed transvaginal surgery, with nearly 40% of the group performing transanal procedures. Most of the participants (88%) worked in a multidisciplinary pelvic floor center.

### General aspects of the role of robotic assistance for PFRS

The first set of practice questions explored general aspects of the use of robotics for PFRS. The experts strongly agreed that the use of the robot to perform PFRS is safe. In general, the group believed that the robotic platform allows better vision during PFRS and that the range of motion and articulation of the robotic instruments provides a specific advantage during this surgery.

The experts agreed that performing PFRS with the robot causes less physical strain and less fatigue for the surgeon compared with conventional laparoscopic surgery. They also thought that the Da Vinci Xi platform reduces operative time versus standard laparoscopy for reconstructing the pelvic floor in complex cases. Nonetheless, they did not feel that using the robot allows the performance of a higher number of procedures within the same day (compared with standard laparoscopy).

The experts also agreed that the learning curve of PFRS procedures is shorter for robotic surgery than for laparoscopy and that it is easier to transfer surgical competencies to other surgeons using the robot. To this extent, the experts subscribed to the idea that having a dedicated center for robotic surgery with expert staff would facilitate use of the robot for non-oncologic applications such as PFRS and that training sessions for inexperienced surgeon assistants are important to reach proficiency.

The experts generally agreed that the robot facilitates quicker discharge of patients. In addition, there was a generalized opinion that collecting patient-reported outcome measures and/or patient-reported experience measures will be a useful tool to show the multidimensional (e.g., socioeconomic) benefits of this technology.

Overall, the experts strongly concurred that they would recommend the use of robot-assisted surgery for PFRS to other colleagues.

### Indications of robotics for PFRS

The second set of practice questions tested the opinions of the experts on the correct indications for the use of the robotic platform for PFRS procedures.

The panel agreed that robotic surgery is preferable to standard laparoscopy for obese patients and for patients with previous abdominal surgeries. Cases of advanced or multicompartmental prolapse, relapsed/recurrent prolapse, or previous reconstructive surgery with meshes were also considered indications for preferring use of the robot over standard laparoscopy. Although the experts concurred that the use of robotics is preferable in more complex cases, they did not believe that robotics should be limited to selected indications.

### Surgical technique in robot-assisted PFRS

The third set of practice items addressed the technicalities of using the robot to perform PFRS.

The experts agreed that the robotic platform provides specific technical advantages over standard laparoscopy for performing all the key steps of abdominal PFRS. Specifically, experts perceived an advantage for dissection of the sacral promontory, the vesicovaginal space, the rectovaginal space, and the Retzius space. In contrast, the experts did not feel that the use of robotics decreases the risk of nerve injury at the level of the promontory compared with standard laparoscopy.

Experts considered suturing in all the districts that are relevant for PFRS procedures to be significantly easier using the robot, including the anterior and posterior vaginal wall, the Retzius space, and the sacral promontory. Suturing on the anterior rectal wall was considered both easier and safer with the robotic technique compared with laparoscopy. Further, mesh placement was thought to be more precise with the use of robotic instruments compared with standard laparoscopy.

Related to safety, the experts did not feel that major vascular damage at the level of the sacral promontory would be any more difficult to manage with robotics than with standard laparoscopy.

On a more technical note, the group agreed that use of the fourth robotic arm is not necessary to perform PFRS in simple cases and that standard robotic monopolar and bipolar instruments are sufficient. The experts expressed interest in the development of a single-port robotic platform for PFRS.

### Future perspectives on robot-assisted PFRS

When the experts were asked about the future role of robotics in the management of pelvic floor defects, they agreed that they would recommend activation of a robotic program for the management of these disorders. The group also deemed it important from the patient’s perspective that a hospital managing pelvic floor diseases is equipped with a robotic platform.

From a health economics point of view, considering both the costs and benefits, the experts agreed that robotic surgery should be considered a suitable technology for patients requiring abdominal correction of POP. The experts also thought that the expanding use of the robot for PFRS may offer socioeconomic advantages (e.g., increased performance of transabdominal reconstructive procedures or possible decreases in relapses).

When asked to select possible refinements of the robotic platform that could improve the performance of PFRS, most experts indicated the development of tactile feedback, artificial intelligence or deep learning dedicated to PFRS, and tools for preoperative anatomical planning and surgical navigation.

### Subanalyses

The results were reanalyzed to explore whether surgeons of different specialties, experience, caseloads, and patterns of surgical practice may differ in opinion on selected questions. Items for which statistically significant differences were found are reported.

Gynecologists strongly believed that pelvic floor reconstruction is a suitable indication for robotics only for selected patients, whereas colorectal surgeons and urologists did not (Table 4).

**Table 4:** Subanalysis by Specialty, Case-Load (Number of Procedures per Year), and Years of Experience (*N* = 26)

Agree	Specialty	Colorectal (*n* = 11)	Gynecologist (*n* = 11)	Urologist (*n* = 4)	*P* value
*Taking into consideration the costs and benefits of RS, PF reconstruction is a suitable indication for robotics only for selected patients*					
No		8 (73%)	2 (18%)	3 (75%)	0.030
Yes		3 (27%)	9 (82%)	1 (25%)	
Procedures per year		< *20 (n* = *11)*	*20–40 (n* = *8)*	> *40 (n* = *7)*	
*The robot allows management of major vascular damage at the level of the sacral promontory*					
No		9 (82%)	5 (62%)	1 (14%)	0.006
Yes		2 (18%)	3 (38%)	6 (86%)	
*It is not necessary to have expertise in traditional laparoscopy to perform robotic PFRS*					
No		6 (55%)	6 (75%)	7 (100%)	0.034
Yes		5 (45%)	2 (25%)	0 (0%)	
*After a day of RS, I feel less tired than after a day of conventional surgery*					
No		5 (45%)	2 (25%)	0 (0%)	0.034
Yes		6 (55%)	6 (75%)	7 (100%)	
Years of Experience		≥ *5 (n* = *20)*	*1–4 (n* = *6)*		
*After a day of RS, I feel less tired than after a day of conventional surgery*					
No		3 (15%)	4 (67%)		0.028
Yes		17 (85%)	2 (33%)		

Data are expressed as frequencies and percentages. Data analysis was done with Fisher’s exact test. Procedures by year analysis was done with chi-square test for trend. Values of *P* < 0.05 were considered significant

*PF* pelvic floor, *PFRS* pelvic floor reconstructive surgery, *RS* robotic surgery

When the opinions of surgeons were analyzed by years of experience (< 5 vs ≥ 5 years), only the more-experienced surgeons agreed that after a day of robotic surgery they felt less tired than after a day of conventional surgery (Table 4).

The opinions of experts were also compared by case-load of robotic PFRS procedures (< 20, 20–40, or > 40 per year) (Table 4). Surgeons performing fewer than 20 robotic cases per year did not feel that the robot facilitates managing major vascular damage at the level of the sacral promontory. Surgeons with a high case-load had an opposite opinion. Surgeons performing more than 40 procedures per year disagreed with the view that it is not necessary to have expertise in traditional laparoscopy to perform robotic pelvic floor surgery. Only the surgeons with the higher case-load agreed that they felt less tired after a day of robot-assisted PFRS than after conventional surgery.

While all the experts were skilled laparoscopic surgeons with experience in laparoscopic PFRS, a subgroup reported currently using only the robot to perform these procedures. We then ran a subanalysis comparing the opinions of surgeons who perform PFRS only with the robot compared with surgeons who perform both robotics and conventional laparoscopic procedures (Table 5). Experts performing only robotic surgery felt that the robot offers several advantages over laparoscopy: decreased rates of complications, more precise and safer isolation of the sacral promontory, easier deep dissection between the rectum and the vagina, better positioning of meshes, easier suturing of meshes on the anterior rectal wall, lower risk of injuries to the pelvic ureters, and easier performance of hysterectomy in the context of PFRS. Experts performing both robotic and laparoscopic surgeries did not agree on the previous points. Only surgeons who exclusively use the robot felt that PFRS is a suitable indication for robotics for every patient.

**Table 5:** Subanalysis by patterns of surgical practice

Agreement	No LPS (*n* = 11)	LPS (*n* = 15)	*P* value
*RS could decrease the rates of intra- and postoperative complications associated with PF reconstruction*			
No	2 (18%)	10 (67%)	0.021
Yes	9 (82%)	5 (33%)	
*Isolation of the sacral promontory is more precise and safer with the aid of the robot*			
No	0 (0%)	8 (53%)	0.036
Yes	11 (100%)	7 (47%)	
*The robot simplifies deep dissection between the rectum and the vagina*			
No	0 (0%)	6 (40%)	0.024
Yes	11 (100%)	9 (60%)	
*The robot allows for better positioning of meshes*			
No	1 (9%)	9 (53%)	0.014
Yes	10 (91%)	6 (47%)	
*Suturing mesh on the anterior rectal wall is easier with the robot than with standard LPS*			
No	0 (0%)	6 (40%)	0.024
Yes	11 (100%)	9 (60%)	
*The robot decreases the risk of injuries of the pelvic ureters*			
No	4 (36%)	12 (80%)	0.043
Yes	7 (64%)	3 (20%)	
*The robot simplifies the performance of hysterectomy in the context of PFRS*			
No	3 (36%)	11 (73%)	0.045
Yes	8 (64%)	4 (27%)	
*Taking into consideration the costs and benefits of RS, PF reconstruction is a suitable indication for robotics for every patient*			
No	1 (9%)	10 (67%)	0.005
Yes	10 (91%)	5 (33%)	

Surgeons performing robotics only (no LPS) vs surgeons performing robotics and laparoscopy (LPS) (*N* = 26). Data are expressed as frequencies and percentages. Data analysis was done with Fisher’s exact test. Values of *P* < 0.05 were considered significant

*LPS* laparoscopy, *PF* pelvic floor, *PFRS* pelvic floor reconstructive surgery, *RS* robotic surgery

## Discussion

Robots are expensive tools that facilitate the performance of surgical maneuvers through better vision and more effective instruments. These technologically advanced platforms ease the execution of minimally invasive procedures by less-experienced surgeons or make the performance of a technically challenging procedure more effective in the hands of an expert laparoscopic surgeon, thus extending the indications of minimally invasive surgery beyond the boundaries set by the limitations of standard laparoscopic instruments [3].

Whether a robotic approach is of significant advantage for a specific procedure depends on the procedure itself, the patient’s characteristics, and the surgeon’s skills; hence it is difficult to measure objectively. If one adds the economic impact of the use of a robot, some procedures, including PFRS, are more debatable as indications because they are associated with lower reimbursements [12, 13].

Nonetheless, the advantage of a surgical tool should be measured based on the degree of technical advantage that it confers during a specific procedure rather than on its cost [16]. The selection of procedures that benefit most from the added cost of such instruments is considered good clinical practice.

Applying the same metric to the complexity of robotic platforms is not easy because multiple parameters influence decision-making. For instance, patients may be attracted toward robotic centers because they consider them more modern [17, 18], which may lead to an economic advantage for the organizations that are equipped with these instruments.

One of the ways to assess the value of robotics in a specific surgical setting is to assess the opinions of expert users who can express through personal experience the technical and nontechnical strengths and weaknesses in comparison with other approaches. We followed this approach to explore the value of robotics in PFRS with the use of a Delphi methodology. This approach has recently become popular to assess robotic surgery [19, 20], including training [21], and the role of innovations related to this surgical approach [22].

Our study confirms that surgeons who use the robot extensively consider this approach safe and generally more effective than standard laparoscopy for PFRS. The experts involved in the Delphi process agreed that the technical advantages of the Da Vinci® robotic platform are evident in all the surgical steps required to perform urogynecology and colorectal PFRS procedures. Specifically, dissection of relevant anatomical planes, mesh placement, suture placement, and knot tying are easier and more effective using the robot than with standard laparoscopy. The participating experts believe that these features allow easier and more effective learning of complex prolapse procedures, particularly in the presence of a robotic center, consistent with previous observations [23–25]. The experts generally agreed that robotics is particularly useful for the treatment of more complex prolapse cases.

Enhanced efficacy and safety were also perceived by the experts; hence they considered the robotic platform as a means to facilitate early discharge of patients. Consistent with this view, recent research has investigated the possibility of enhanced recovery after surgery protocols combined with early, same day, and even ambulatory surgery for PFRS procedures performed with the robot [26–30].

Some interesting differences were identified in the group subanalyses. Gynecologists felt that robotic POP surgery should be limited to selected and more complex cases, whereas urologists and colorectal surgeons did not. This difference could be due to the different case mixes of procedures and selection algorithms for robotics in the 3 specialties; however, since urologists were less numerous in the expert groups, this finding might be biased.

A number of experts over time had chosen to perform PFRS only with the robot. This group showed a stronger appreciation for the technical advantages of this platform compared to those who used both robotics and standard laparoscopy. They indeed believed that robotics should not be limited to selected cases but that it is instead useful also for less complex procedures. This finding shows how within an expert group of pelvic floor surgeons, a significant proportion elected using only the robot to perform PFRS due to perceived technical advantages. We did not explore whether those using both laparoscopy and robotics did so for a strategic surgical choice or because of other factors (e.g., limited access to the robotic platform).

Another interesting difference was that the more-experienced surgeons were more likely to emphasize the ergonomic advantages of the robotic platform and were convinced that it decreased the duration of PFRS procedures, possibly indicating that continued use leads to better exploitation of the benefits of the robot. This may suggest that centralization of robotic procedures in the hands of high-volume surgeons could have advantages.

Overall, the general feeling of the group of gynecologists, urologists, and colorectal surgeons’ expert in the use of the robotic platform for PFRS was that robotics is appropriate for this type of surgery given the significant technical advantages and the overall cost–benefit balance. This Delphi initiative calls for more research to objectively assess the specific settings where robotic surgery might be more wisely chosen over standard laparoscopy, with the overarching objective of improving patient care and sustainability.

### Study limitations

This study has some limitations. The study population is a self-selected group of expert pelvic floor surgeons who may have more positive feelings and confidence with robot-assisted surgery than those who did not participate. Also, while the sample size of the total group is within the recommended range for a Delphi analysis, groups in the subanalyses are smaller, which is an obvious limitation.
